# Home-Based Care Type II Workplace Violence Nurse Provider Experience: A Literature Review

**DOI:** 10.1097/NHH.0000000000001407

**Published:** 2026-01-07

**Authors:** LaDawna Goering, Tracy Ashby

**Affiliations:** **LaDawna Goering, DNP, APRN, ANP-BC, BC-ADM, CDP,** The University of Texas Health Science Center at Houston, Cizik School of Nursing, Houston, Texas.; **Tracy Ashby, MLIS,** is a Research & Instruction Librarian, Texas Medical Center Library, Houston, Texas.

## Abstract

Type II workplace violence (WPV)—violence committed against healthcare workers by patients, clients, or visitors—poses a significant threat to home-based care providers. Due to the unique, isolated nature of home care, these providers are particularly vulnerable, often lacking home-specific training and safety resources. A systematic review of literature published between 2014 and 2024 was conducted to examine qualitative and quantitative studies addressing Type II WPV in U.S. home health settings. Eight studies met the inclusion criteria. The review revealed that WPV in home care is frequently underreported, with existing training programs primarily designed for hospital environments rather than private homes. As a result, home care providers face heightened risks of both physical and psychological harm. The studies consistently called for specialized, home-based WPV prevention training emphasizing patient screening, situational awareness, and de-escalation strategies for high-risk clients. Future research should focus on developing and implementing comprehensive, home-specific WPV prevention and reporting protocols, alongside supportive organizational policies that promote a culture of safety and accountability for home healthcare workers.

**Figure:**
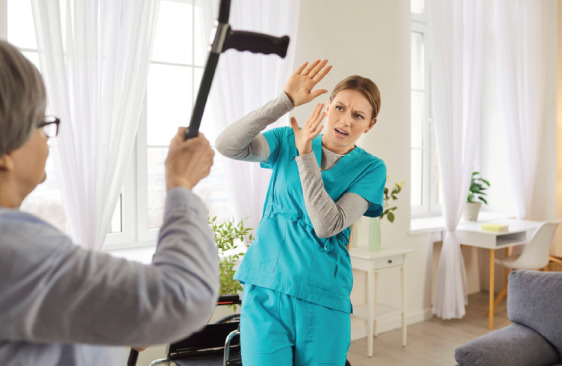
No caption available.

The home healthcare industry provides highly specialized and personalized care to individuals who prefer to receive treatment in the comfort of their own homes. In 2021, approximately 3 million Medicare patients received home-based care, and this demand is expected to continue growing (Centers for Medicare & Medicaid Services [CMS], 2024). Acute and long-term care facilities increasingly depend on home care services to transition patients out of hospitals, reducing overall healthcare costs, minimizing long-term care expenses, and maintaining hospital bed availability (CMS, 2020). The home healthcare market is projected to grow by 33% between 2020 and 2030 (Bureau of Labor Statistics [BLS], 2021). However, this rapidly expanding industry also faces escalating workforce risks, one of the most significant being Type II workplace violence (WPV), violence committed against healthcare workers by a patient, client, or visitor in the context of their work (CDC, 2024).

WPV is a recognized hazard across all healthcare professions. Although statistics on WPV can be challenging to pinpoint owing to variations in sampling methodologies, studies consistently indicate an upward trend (US Department of Justice, 2022). Nurses are at a significantly higher risk than other professions due to the personal nature of care and the environment and sociocultural context in which patient interactions occur. In 2020, healthcare and social assistance workers experienced a high incidence rate of WPV injuries at 10.3 per 10,000 incidents, with nurses reporting an even higher rate of 21.8 per 10,000 (BLS, 2021).

WPV in healthcare has garnered significant attention, and risk factors for violence vary considerably between hospital and home care settings. Most WPV prevention programs are designed for inpatient environments, neglecting the vulnerabilities of home care providers. Of all reported WPV incidents, 73% involve healthcare workers, and 58% occur in limited-access areas, primarily within patients' homes (BLS, National Institute for Occupational Safety and Health [NIOSH], 2022). Many home care providers lack the training to handle WPV; only half of home care workers reported receiving WPV prevention training upon being hired (Balkaran et al., 2024). According to the U.S. BLS (2018), home healthcare providers have a fivefold higher risk of WPV compared to individuals working in other industries. Furthermore, the U.S. BLS (2018) reported that healthcare workers experience violent assaults at a rate four times higher than workers in other industries; for nurses and other personal care workers, this rate is 12 times higher. Nurses often develop close personal and emotional relationships with patients, rendering them particularly vulnerable to WPV (Zhang et al., 2023). Physical violence against nurses has been reported to occur at twice the rate of such incidents against physicians, and emotional WPV is also significantly more prevalent among nurses (2023). WPV against nursing providers has significant physical and psychological consequences, ranging from minor injuries to severe long-term effects. In addition to immediate trauma, nurses often experience feelings of guilt, fear, anxiety, depression, stress, burnout, and diminished morale, which can result in decreased productivity, increased absenteeism, and, in some cases, job resignation (Lucien et al., 2024). Furthermore, WPV directly compromises the quality of patient care by leading to shortened visits, altered provider responses, and disruptions in care coordination (Möckli et al., 2020). These effects have broader implications for healthcare economics, such as increased employee absences, high staff turnover rates, and a shrinking workforce. Ultimately, these challenges disrupt the continuity and quality of care transitions and exacerbate the strain on healthcare systems (Möckli et al., 2020).

## Definition of WPV/Type II Violence

This literature review examines Type II WPV, as defined by national and international organizations. The World Health Organization (2025) categorizes violence and harassment as work-related abuse, threats, or assaults that include physical, sexual, verbal, and psychological abuse, as well as workplace harassment. The Centers for Disease Control and Prevention (CDC) and the National Institute for Occupational Safety and Health (NIOSH, 2024) classify four types of WPV.

- Type I involves a perpetrator without a relationship to the assault or violence and occurs less frequently than the other types.
- Type II WPV is an assault perpetrated by clients or customers against workers where there is a relationship, referred to as client-on-worker violence. It is the most frequent form of WPV in healthcare settings, frequently occurring in emergency departments, psychiatric facilities, waiting rooms, and geriatric care environments, and is the focus of this literature review.
- Type III violence occurs among coworkers at a lateral or horizontal level, often referred to as bullying, or worker-on-worker violence.
- Type IV is an outside violence that happens to occur in the healthcare environment, for example, a personal environment that carries into the workplace.

Given that Type II WPV is most frequently encountered by home care nurses, this review explores the prevalence, impact, and mitigation strategies related to Type II WPV.

## Definition of Home Healthcare/Home Health Nursing

The authors acknowledge recent changes in terminology surrounding home healthcare, which encompasses a diverse range of services, including home-based primary care, hospital-at-home care, home-based acute care, and specialized services. The American Academy of Home Care Medicine (2022) defines home health and home care as umbrella terms that include these specialized home services. This review incorporates nursing studies that use varied terminology for home care to provide a comprehensive understanding of the field.

## Current Guidelines on WPV Prevention

In 1970, the Occupational Safety and Health Act (OSHA) established NIOSH to research and promote safe, healthy workplaces. NIOSH offers three courses on WPV prevention: one focuses specifically on nurses, and the other addresses inpatient environments. The guiding principle for these courses emphasizes a multifactorial approach that includes education as both a preventive and intervention strategy (NIOSH, 2024). According to the U. S. Department of Labor, OSHA Guidelines for preventing WPV (USDOL/OSHA 2016), several adaptations have been proposed for safety in home healthcare settings, including engineering controls, vehicle maintenance, improved lighting, Global Positioning System tracking, logging systems, and entrance/exit route assessments. However, there are no specific guidelines or safety competencies tailored to the unique needs of home healthcare providers and violence prevention. The Joint Commission (2022) mandates hospital and critical access hospital programs and behavioral health, requiring multidisciplinary teams to develop policies and procedures for preventing, responding to, reporting, and following up on WPV incidents. Unfortunately, these programs do not address the safety concerns of home care providers. Similarly, the American Organization for Leadership and the Emergency Nurses Association has created a toolkit for mitigating WPV (2022). Although this toolkit guides the development and evaluation of WPV prevention programs, it does not explicitly target the challenges faced by home healthcare workers. Reviewing existing evidence and publicly available resources reveals a clear gap in WPV training specific to home care providers.

## WPV in the Home Care Setting

This review postulates that current recommendations, curricula, and training do not adequately reflect the dangers encountered in home healthcare settings or prepare nursing providers for these challenges. Although literature reviews on this topic exist, most are limited in scope and relevance. Most studies are more than a decade old or focus on international settings.

The objective of this literature review is to provide a comprehensive understanding of Type II WPV in home-based care. The review focuses on the experiences of nurse providers, the adequacy of training, and the effectiveness of reporting practices within the United States.

## Methods

A systematic search to compile literature on studies reporting on WPV prevention, safety, and policies for nurses in a home care environment was conducted using MEDLINE on the Ovid platform as the primary database; the topic and the following related concepts were explored: home care, nurses, workplace violence, safety, and policy. The ideas were developed using both controlled and natural languages within Ovid MEDLINE. MeSH terms were identified, and keywords, along with their synonyms, were gathered. The keywords were searched using the Ovid MEDLINE database's title, abstract, and keyword fields. The results were limited to the past 10 years (January 1, 2014–December 31, 2024). The search strategy was then translated from MEDLINE (Ovid) to Embase (Elsevier), CINAHL Plus with Full Text (EBSCO), and Web of Science (Clarivate; see Supplemental Appendix A at http://links.lww.com/HHN/A188). The results were uploaded to the EndNote citation manager, and de-duplication was completed within the EndNote 20 citation management system and performed manually. Before de-duplication, citations totaled 99; after de-duplication, the total number of citations was 54.

## Inclusion and Exclusion Criteria

**Figure 1.**
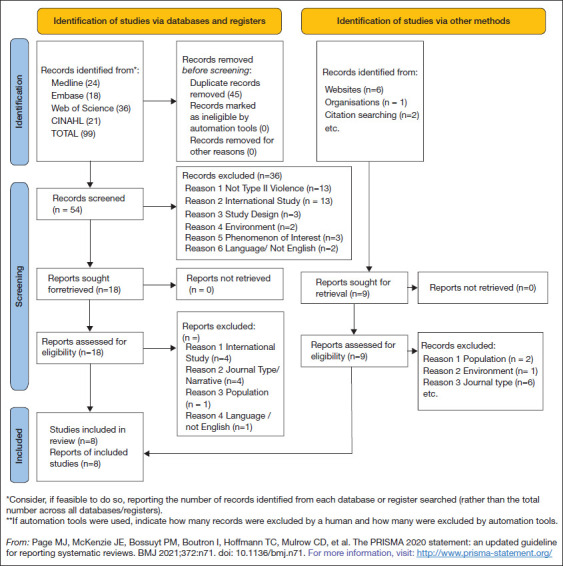
PRISMA Flow Diagram of Studies.

Records were included if they were (1) conducted primarily in the United States, (2) written in English, (3) published in peer-reviewed journals, (4) research-based, (5) published within the past 10 years, (6) focused on home-based care WPV toward nurse providers (Type II WPV), and (7) categorized as knowledge inquiry or intervention studies. Additional articles were identified through data mining from articles retrieved from database searches (see Figure 1).

## Results

The authors reviewed the titles of 54 identified studies, resulting in the removal of 36 articles: 13 did not address Type II violence, 13 were international studies, three were excluded due to study design (commentary), two were unrelated to the home care setting, three focused on different phenomena (e.g., burnout or stress), and two were not in English. Additional searches of websites, organizations, and data mining yielded nine articles. Two were excluded due to population (not Type II WPV), one for the environment, and six for study or journal type. Subsequently, 18 journal abstracts were retrieved and reviewed by three independent reviewers. Of these, one was excluded for being an international study, four for journal type, one for population focus, and one was not in English.

### Characteristics of the Included Studies

The initial search included international studies; however, the team decided to focus on American studies, due to the variations in home healthcare between the United States and other countries, as well as the culture of home-dwelling and international communities. Eight studies and reviews were selected, published between 2017 and 2024. The studies were primarily conducted and published in the United States; international reviews were only included when the United States studies were the primary location of study, or they were excluded as predominately international-based study reviews, two scoping reviews (Balkaran et al., 2024; Schnelli et al., 2023) and one systematic review (Lucien et al., 2024) were included based on this criterion. Two studies were cross-sectional (Semeah et al., 2017; Small et al., 2022), two were mixed-methods studies (Byon et al., 2020; Karlsson et al., 2019), and one study was a data analysis (Byon et al., 2023). Studies ranged from small studies at an independent agency to large home health agencies and included an extensive database study (see Table 1).

**Table 1:** Workplace Violence Prevention/Safety Evidence Table

Citation/Source	Study Design/Method	Setting/Population	Aims/Objectives	Results/Clinical Relevance
Balkaran et al. (2024)	Scoping Review Databases: Medline, EMBASE, OVID, Scopus, EBSCO host 46 inquiry/interventional studies from 2011-2021 28 Quantitative 13 Qualitative 5 Mixed-Method studies	US *n*=21 Canada *n*=4 Australia *n*=1 Finland *n*=1 Germany *n*=2 India *n*=1 Israel *n*=5 Japan=2 New Zealand *n*=1 Norway *n*=1 Norway *n*=1 Rio De Janeiro *n*=1 South Korea *n*=2 Switzerland *n*=4	Map current research on abuse in-home care and interventions	Three themes emerged 1. Prevalence and types of abuse 2. Abuse in the context of living with dementia 3. Working conditions and abuse Risk factors for home care workers to experience abuse younger age temporary position limited home experience limited interaction time with patients Verbal abuse is most common—51% Physical abuse 2.5% Sexual abuse requires police reporting Abuse toward home care workers by clients living with dementia merits separate analysis—increase risks by 145% (behaviors/ risks/ response) Stressful job demands and communication breakdown contribute to a normalized culture of abuse in the home care setting. Evidence of a clear gap, with no concrete examples of WPV HH training/policies Clinical Relevance: Regular Workplace Training improves knowledge, skill, and ability to respond to WPV—action training and coaching sessions proved to be the best sources of education Recommendations for intergenerational system—mentoring on home care visits. Provide supportive environment and peer groups/ (provide resiliency/ self-worth) Provide anonymous reporting guidelines
Byon et al. (2023)	Data analysis research study Machine learning based on natural language processing algorithm applied to 600,000 clinical visit notes— 1,515 notes manually reviewed by MSN RN in HH for Type II WPV EHR data RN clinical home visit notes	US 2 Large Home Health Agencies East Coast	Identifying hidden Type II workplace violence from clinical notes using a natural language processing system	Identified 236 unreported incidents of Type II WPV toward home healthcare nurses Physical violence 222 incidents, 0.067 per 10,000 Nonphysical 20 incidents, 3.76 per 10,000 Any violence 4/ 10,000 During this time period, 0 incidents were reported Clinical Relevance: Language processing can assist managers in identifying unreported WPV & potential violence risks. Incident report systems do not accurately account for the number of WPV incidents/ even if recorded in clinical notes
Byon et al. (2020)	Mixed Methods Quantitative online health nursing survey Qualitative Interview & Focus groups Home health—1.5 hrs	Convenience sample 18 Home Health Nurses 5 management staff = 23 home healthcare providers	To describe the extent of reporting WPN events and to identify barriers and facilitators to reporting Understanding reporting of Type II workplace violence among home healthcare nurses	Home health nurses in the last 12 months (Reported) 5.6% experienced physical violence (100% reported) 33.3% experienced threats of harm (100% reported) 50% verbal abuse (89% reported) Benefits / Disadvantages of Reporting HHNs reported WPV when they felt it benefitted other HHNs / protection for HHN safety / recording the incident and supported by management. Disadvantages / Not reporting Reliving trauma Unstandardized reporting Uncertain of what was reportable (lack of clear definition)—tolerated from certain conditions (dementia/ brain injury)/ patients frustrated about HC Lack of time Part of the job Management perceived the lack of a standardized reporting process most significant barrier to reporting Clinical Relevance: Reporting can be influenced by perceived culture if reporting is perceived as beneficial and supportive or disadvantageous/ discouraged by management. Standardizing the reporting process is needed with a simple process and standard policy that supports staff and follow-up.
Karlsson et al. (2019)	Mixed-Methods Study Questionnaire Survey Responses Risk factors identified by univariate and multivariable analyses	US *N*=954 Home Health Care aides	Examine verbal abuse in large home care aide population and evaluate risk factors	22% of aides reported at least one incident of verbal abuse in the prior 12 months 3 factors / multivariable models Clients with dementia, mental illness, or psychological issues, or limited mobility Homes with confined space Unpredicted work hours 2 additional factors associated with verbal abuse Clients with limited mobility Unclear plan for care delivery Aides who reported verbal abuse were 11 times as likely to report physical abuse. Clinical Relevance: Training for specific population groups and communication strategies is needed, as are predicted work hours and care plans for patients, with home assessments (assess confined spaces and safety)
Lucien et al. (2024)	Systematic Mixed Studies Review (*n* = 10) were quantitative nonrandomized studies, and the others were focus groups (*n* = 1) and mixed methods (*n* = 1) 3 Electronic Databases: EBASE, CINAHL, PsychINFO PRISMA-ScR *N*=12 7 cross-sectional 4 interviews/survey 1 documentation analysis	US *n*=10/ Switzerland =2	Prevalence of violence by home care recipients against caregivers and types and consequences of violence. (1) What are the different types of WPV in the home care setting? (2) What is the prevalence rate of WPV toward formal and informal caregivers by care recipients in the context of home care? (3) What are the consequences of WPV for formal and informal caregivers? (4) What is the context for formal caregivers experiencing WPV? (5) What is the context for care recipients who inflict WPV?	Experienced WPV during last 12 months 7.9%–61.3% 2.5%–27.5% experienced physical violence 12.8% experienced sexual aggression/ 27.6% sexual harassment, 2.3% were kissed in a way that made them feel uncomfortable, and 0.3% of them were raped Consequences of WPV Negative psychological reactions (depression/stress/sleep problems/ burnout/ PTSD) that could lead to a reduction in the quality of care (absentee/ decreased pt time) and staff turnover Risk factors 1. younger age 2. less experience 3. less frequent visits 4. less home care experience 5. non-predictable hours 6. lack of care plan 7. limited home space 8. Patient with mobility limitations or mental illness Clinical Relevance: WPV in the home is underestimated, WPV in the home does not represent the hospital setting. Targeted adaptation of prevention strategies is needed to reduce WPV in the home. In-home care in the private sphere of care recipients is much more complicated to collect and track these events.
Schnelli et al. (2023)	Scoping Review CINAHL PubMed Cochrane WOSCCC PsycINFO PRISMA-ScR 7 journals/6 Grey literature Qualitative/ Mixed methods/ Experimental/ OSHA/ Theoretical frameworks	11 US 4 International (1 South Korea/3 Japan)	Examine current research and grey literature on aggressive incidents of persons with dementia toward professional caregivers in the home setting	4 Themes Emerged (1) Aggressive Behavior—PLWD risk factor due to confusion impairment in cognition (2) Triggering Factors: Expression of need and caregiving activities (3) Lack of Skills & Educational Needs Related to PLWD (4) Hindrances to Problem Solving of Aggressive Behavior: fear, underrecognizing aggressive behavior & sense of duty, and lack of monetary or structural resources Clinical Relevance: Need for dementia-specific education, regarding communication skills, aggression triggers, and responding to aggressive behavior is needed for this population group. Lack of reporting of incidents related to PLWD has a clear relationship to solving aggressive behavior patient needs and under recognition of dementia behaviors and lack of resources.
Semeah et al. (2017)	Cross-sectional Pilot study—37 item survey study	HUD/ VASH Supportive Housing- recipients of home-based services to veterans' homes (homeless or previously homeless population) North Florida South Georgia Veterans' Health System HUD/VASH 35 surveys	Examine how safety concerns act as a barrier to services and optimal patient outcomes Develop a baseline of violence and aggression frequency and reporting toward Federal healthcare workers in the HUD-VASH program	88.6% experienced verbal abuse 88.5% experienced patient violence/ aggression 85% experienced violence 80% verbal abuse 8.6% sexual abuse 34.3% reported reporting the incident 54.3% did not report 17.1% experienced physical abuse 70% of HHCW exposed to abuse/ 54% were not reported Clinical Relevance: Providing services to homeless veterans warrants further examination for the protection of workers against workplace violence, where the VA flagging system of identifying patients with aggressive or disruptive behaviors / has a different need to provide for home-based care.
Small et al. (2022)	Cross-Sectional Design Violence against home healthcare and hospice workers survey	US Ohio 2 HHC agencies 50 HHCWs HHC Workers: APNS, PAs, SW	Describe workplace violence prevention training, resources, and commitment to HHC safety	62.5% offers WPV training 48.9% received WPV prevention training 95.7% reported training on reporting 82.6% on aggressive behavior 78.3% on customer service 77.3% on procedure on seeking medical care 45.5% on psychological care 33.3% had an escort or buddy system 65.2% contained information on hazardous neighborhoods 43.97% utilized cell phones as a safety resource (2.3% were employer provided) Clinical Relevance: Only half received WPV training/need for specific home-based training/culture of safety and zero tolerance toward WPV

### Experience of Type II WPV

Research findings highlighted nursing providers' experiences with Type II WPV in home care settings, focusing on its incidence, associated risk factors, and consequences. Across all reviewed studies, the prevalence of WPV was significantly underreported. Researchers also examined WPV preparedness as part of the overall experience, except for Byon et al. (2023), whose data analysis focused solely on incident documentation. Several studies pointed to the normalization of Type II WPV, as “part of the job” within the home care environment in which they are a patient's “guest,” which contributed to its persistent underreporting and increasing prevalence (Balkaran et al., 2024; Byon et al., 2020; Lucien et al., 2024). Despite this normalization, feelings of insecurity, fear, anger, chronic anxiety, and depression, as well as decreased job satisfaction and performance, are reported by care providers who have experienced WPV (Balkaran et al., 2024; Byon et al., 2020; Lucien et al., 2024). This cultural acceptance of violence in home healthcare highlights a critical barrier to addressing and mitigating WPV effectively.

### Incidence of Type II WPV in Home-Based Nursing

The studies revealed a prevalence of WPV in home care settings with verbal abuse as the most common form, ranging from 22% to 80% (Balkaran et al., 2024; Byon et al., 2020; Karlsson et al., 2019; Lucien et al., 2024; Schnelli et al., 2023; Semeah et al., 2017), occurring in just over 51% of cases. This category included behaviors such as yelling, screaming, and insulting. Although physical 2.5% to 85% (Balkaran et al., 2024; Byon et al., 2020; Karlsson et al., 2019; Lucien et al., 2024; Schnelli et al., 2023; Semeah et al., 2017) and sexual abuse 8.6% to 27.6% (Lucien et al., 2024; Semeah et al., 2017) occurred, it was found to be reported less frequently; providers often felt compelled to either resign or escalate the matter to law enforcement (Balkaran et al., 2024). Semeah et al. (2017) reported the highest prevalence of WPV, with 70% of home care providers experiencing some form of violence. Alarmingly, 54% of these incidents went unreported.

### Risks Related to Type II WPV in Home-Based Nursing

Provider characteristics and situational factors significantly influence the risk of WPV. Younger age, limited experience in home environments, and insufficient violence prevention training increase providers' vulnerability to Type II WPV (Balkaran et al., 2024; Karlsson et al., 2019; Lucien et al., 2024). Additionally, limited patient interaction time and temporary positions further exacerbate WPV risks (Balkaran et al., 2024).

Patient-related risk factors, particularly those involving physiological or psychological crises, can heighten the likelihood of violent behavior toward healthcare providers. Conditions such as infections, inflammation, pain, and delirium can alter a patient's mental state, lowering the threshold for aggression. These challenges are particularly pronounced in home care settings, where oversight and rapid intervention options are often limited (Lucien et al., 2024).

Environmental factors within home settings also affect WPV risk, and confined or restricted spaces, such as small rooms or cluttered environments, can present unique challenges for healthcare providers. The physical layout of a patient's home and mobility limitations can heighten the risk of aggression or bodily harm to workers (Balkaran et al., 2024; Karlsson et al., 2019; Lucien et al., 2024). Time-related factors were also found to influence WPV risk: clear work plans reduced the likelihood of WPV, whereas the absence of structured planning increased its occurrence (Karlsson et al., 2019). Specific patient populations present a higher risk of WPV toward healthcare providers, particularly in home care settings. These groups, which include veterans and individuals living with dementia, mental illness, substance use disorders, limited mobility, and homelessness, face unique medical, psychological, or social challenges that increase the complexity of interactions. Specialized education in communication and responding to these patients' specific needs and behaviors is needed to help healthcare providers effectively care for these high-risk populations while recognizing and responding to aggressive behaviors that may prevent WPV incidents (Balkaran et al., 2024; Karlsson et al., 2019; Lucien et al., 2024; Schnelli et al., 2023).

## Consequences of TYII WPV

Type II WPV has been associated with significant adverse psychological effects, including depression, fear, anxiety, burnout, and post-traumatic stress disorder, as well as physical injuries (Lucien et al., 2024; Schnelli et al., 2023). These effects can impair job performance, strain provider–patient relationships, and reduce the quality of care. Additionally, patient visit times may decrease owing to these challenges (Lucien et al., 2024). The economic impact of WPV on home care is substantial, as it contributes to high turnover rates and absenteeism, further disrupts the continuity of care, and places additional strain on the workforce (Lucien et al., 2024).

## WPV Training

Studies examining WPV identified one of the most significant risk factors for Type II WPV as the lack of home-based WPV training and education (Balkaran et al., 2024; Small et al., 2022). The most relevant study related to WVP training research was conducted by Small et al. (2022), who found that only half of the participants had received WPV training, and the content was limited to managing aggressive patients. Additionally, the training lacked critical components such as patient pre-screening, de-escalation techniques, hands-on training, and violence prevention strategies, which are vital elements to preventing WPV. Balkaran et al. (2024) observed that regular WPV training significantly improved providers' knowledge, skills, and ability to respond to Type II WPV; furthermore, the review found that regular coaching sessions improved home care workers' ability to protect themselves from abusive interactions by ending home care sessions early. The review highlighted the critical need for a gap in the availability of concrete, as well as access to home-specific WPV training programs and policies (Balkaran et al., 2024). Across all reviewed studies, researchers emphasized the importance of home-based WPV training combined with supportive reporting guidelines for effective WPV prevention. These findings underscore the need for tailored training that equips home care providers to handle the unique challenges of WPV in nontraditional healthcare environments, which can be unpredictable (Balkaran et al., 2024; Byon et al., 2020; Karlsson et al., 2019; Lucien et al., 2024; Schnelli et al., 2023; Semeah et al., 2017).

### Reporting of WPV in Home-Based Care

The home care profession has culturally accepted underreporting of WPV. In a study examining clinical notes, Byon et al. (2023) found 236 WPV incidents charted with zero reported incidents. Providers have offered several reasons for this underreporting, including a lack of time, a desire to not relive the trauma, a faulty reporting system, a lack of leadership support, and acceptance of WPV as “part of the job” (Byon et al., 2020). Lucien et al. (2024) also noted the difficulty of tracking WPV owing to the private sphere of home care, which complicates reporting collection. However, reporting was increased when it was felt to benefit other providers or viewed as protective and supported by leadership in psychological safety (2020). These concepts are also felt to be part of the experience of WPV in the home environment, which can be different than the hospital environment due to the autonomous nature of the work. Byon et al. (2020) identified several key benefits of reporting Type II WPV, including the ability to monitor trends for patterns of violence, identifying patients to prevent occurrences, and providing support and recovery for healthcare workers. Additionally, establishing standardized clinical language for nurses to describe Type II WPV can assist healthcare leadership in identifying, documenting, and tracking risk factors that contribute to provider safety (2020).

## Special Populations of People

Most studies, with one exception, identified population groups at increased risk of Type II WPV in the home care environment. These groups include individuals with mental illness, dementia, limited mobility, a history of alcohol or substance use, or those experiencing homelessness (Balkaran et al., 2024; Byon et al., 2020; Karlsson et al., 2019; Lucien et al., 2024; Schnelli et al., 2023; Semeah et al., 2017). Each of these populations has unique vulnerabilities and behavioral characteristics that increase the likelihood of violent incidents, particularly in the isolated and less controlled settings of home healthcare. Several studies specifically identified persons with dementia as an increased risk to home care nursing providers and noted a need for increased training and research (Balkaran et al., 2024; Byon et al., 2020; Karlsson et al., 2019; Schnelli et al., 2023). Balkaran et al. (2024) reported that individuals with dementia exhibited a 145% higher incidence of violent behavior compared to other patient populations. This elevated risk is attributed to the cognitive and behavioral challenges associated with dementia, including confusion, disorientation, memory loss, and decreased impulse control, all of which can lead to aggressive responses. Schnelli et al. (2023) further examined dementia-related aggression, identifying triggers such as unmet patient needs and personal care activities. Their findings underscore the need for specialized training and resources to equip providers to care for this vulnerable population effectively. Semeah et al. (2017) examined the prevalence of violence among home care providers working with homeless veterans, finding that 88.5% of providers had experienced incidents of violence or aggression. This highlights the complex health, social, and behavioral challenges faced by this population, further complicating care delivery and increasing risks for providers.

## Discussion

This literature review on Type II WPV in home healthcare highlights the significant threat WPV poses to nurse providers. The unique vulnerabilities of nurse providers in home settings are compounded by limited access to tailored training and resources. Despite the rising incidence of WPV, many cases remain unreported, exposing a critical gap in awareness and preventive strategies.

Specialized training designed for home care is notably lacking, impeding effective communication and safety measures. Nursing providers, especially those working with high-risk populations such as individuals with dementia, alcohol or substance abuse, or unstable housing conditions, need targeted training to address these distinct challenges.

This review underscores the urgent need for comprehensive research to develop targeted safety protocols for WPV, including reporting procedures and support systems for healthcare providers operating in home settings. Healthcare stakeholders must prioritize developing policies to mitigate WPV risks in home care. Such initiatives should involve a detailed examination of the incidence and classifications of violence in these environments, as well as the identification of high-risk factors unique to home-based care.

Furthermore, additional research is necessary to understand the broader effects of Type II WPV on providers and patients. This includes its impact on care quality and the healthcare system at large. Although several studies have identified high-risk groups within home-based care, only Karlsson et al. (2019) proposed solution-focused training. This highlights a pressing need for more comprehensive educational initiatives and supportive reporting frameworks to safeguard healthcare providers and improve care outcomes.

## Future Research & Implications for Practice

Considering the findings of this review, future research is essential to deepen the understanding of Type II workplace violence (WPV) experienced by home-based healthcare workers. Further investigations should aim to identify risk factors, develop effective prevention strategies, and establish robust reporting and support mechanisms tailored to the unique challenges of home healthcare environments. Addressing these knowledge gaps will enable healthcare systems to design comprehensive policies and implement evidence-based training programs that prioritize nurse safety while enhancing the quality of home-based care delivery.

This review provides home-based nurse providers with a foundation for recognizing risks within the home environment and among population groups where WPV incidents are most prevalent. It underscores the need to advocate for WPV education, standardized reporting procedures, and ongoing professional development. Nurse providers play a pivotal role in transforming the culture of underreporting WPV by promoting physical and psychological safety within the profession and fostering environments of peer and mentor support.
